# Overexpression of TPM4 is associated with worse prognosis and immune infiltration in patients with glioma

**DOI:** 10.1186/s12883-023-03058-0

**Published:** 2023-01-13

**Authors:** Yao Li, Yanan Zhang, Zeyu Wu, Peng Sun

**Affiliations:** 1 Department of Neurosurgery, Qingdao University, Qingdao, 266003 Shandong Province China; 2 Department of Anesthesiology, Weifang Medical University, Weifang, 261053 Shandong Province China; 3 Department of Neurosurgery, Affiliated Hospital of Qingdao University, No.16 Jiangsu Road, Qingdao, 266003 Shandong Province China

**Keywords:** TPM4, Glioma, Overall survival, Prognosis, Immune infiltration

## Abstract

**Background:**

Tropomyosin 4 (TPM4), a member of the tropomyosin family, is aberrantly expressed and plays an important role in a variety of cancers. However, studies on TPM4 in glioma patients are currently lacking.

**Objective:**

Our study aimed to evaluate the diagnostic and prognostic characteristics of TPM4 in glioma and its correlation with immune infiltration.

**Methods:**

Bioinformatic analysis was performed to determine whether TPM4 has diagnostic and prognostic value for glioma. The following databases and analytical tools were used to explore the clinical significance of TPM4 in glioma: TCGA, GTEx, GEO, STRING, and TISIDB.

**Results:**

Our study showed that the mRNA and protein expression levels of TPM4 were significantly higher in glioma than in healthy brain tissue. Kaplan–Meier analysis indicated that high expression of TPM4 in glioma correlated with poor prognosis. Univariate Cox analysis indicated that the high expression level of TPM4 in glioma was an independent prognostic characteristic for low overall survival (OS). The areas under the 1-year survival ROC, 2-year survival ROC, and 3-year survival ROC were all greater than 0.8. GO and KEGG enrichment analysis and GSEA showed that humoral immune response and cytokine receptor interaction were significantly enriched in the TPM4 high expression group, where M phase of the cell cycle, neutrophil degranulation, signaling by interleukins, and signaling by rho GTPases were significantly enriched. Furthermore, according to the analysis of immune cell infiltration, TPM4 was associated with tumor infiltration of a variety of immune cells.

**Conclusions:**

In conclusion, our study suggests that TPM4 may be an effective prognostic biomarker for glioma patients, providing new ideas and research directions for glioma research.

## Introduction

Gliomas, accounting for 75% of primary malignant brain tumors in adults [1], are the most common primary malignant brain tumors. According to the 2016 World Health Organization (WHO) classification, gliomas are divided into four grades: grades I and II are low-grade gliomas (LGG), and grades III and IV (glioblastoma, GBM) are high-grade gliomas [2]. Low-grade gliomas (LGGs) tend to have a better prognosis and a lower degree of malignancy, whereas high-grade gliomas often lead to severe clinical outcomes; however, LGGs can eventually develop into high-grade gliomas [3]. Current treatments for glioma include surgery, postoperative adjuvant chemoradiation therapy, and immunotherapy. Nevertheless, the prognosis of glioma patients is still poor [4]. Hence, it is necessary to identify potentially reliable biomarkers to guide the prognosis and treatment of glioma.

Tropomyosin (TPM) is the main structural component of cytoskeletal filaments, and its family members mainly include TPM1, TPM2, TPM3, and TPM4, which are widely expressed in muscle and nonmuscle cells [5, 6]. Tropomyosin 4 (TPM4) is mainly involved in the contraction of skeletal and smooth muscle cells or maintains the stability of the cytoskeleton in nonmuscle cells and plays a pivotal role in regulating cytoskeletal function and muscle contraction [7]. In the last decade, abnormal expression of TPM4 has been confirmed to be related to the occurrence and development of lung cancer [8], hepatocellular carcinoma [9], pancreatic cancer [10], bladder cancer [11], and breast cancer [12]. However, their correlation with TPM4-related expression patterns, prognostic values, and the microenvironment of glioma tumors remains to be explored.

Here, we aimed to elucidate the expression pattern of TPM4 in whole-grade glioma and its value in the diagnosis and prediction of prognosis in glioma by using The Cancer Genome Atlas (TCGA) and Gene Expression Omnibus (GEO) databases, as well as to determine its relationship to immune cell infiltration and immune checkpoints. Our findings suggest that TPM4 may be a novel and effective biomarker for glioma, offering new hope for improving the survival and prognosis of glioma patients.

## Materials and methods

### Data

We used both the TCGA (https://portal.gdc.cancer.gov/) and the GTEx (https://www.gtexportal.org/home/) (Genotype-Tissue Expression Project) databases to investigate the levels of TPM4 expression in various types of healthy tissues and tumors. The TCGA is available to the public and can be directly accessed, so no local ethics committee approval is required [13]. RNA expression profiles (RNA-Seq2 level 3 data; format: TPM; platform: Illumina HiSeq 2000) and clinical samples were obtained from glioma patients from the TCGA database. The TCGA contains 689 glioma samples and 5 normal brain tissue samples, which include general information, prognostic information, and clinicopathological details. We obtained the gene expression profiling dataset (GSE50161) from the GEO database (https://www.ncbi.nlm.nih.gov/gds). In this study, we used 13 normal samples and 49 glioma samples from GSE50161. Data were analyzed by using R 3.6.3 software.

### Human Protein Atlas (HPA)

Immunohistochemical staining images of glioma and normal adjacent tissues were collected from the HPA (https://www.proteinatlas.org/). The HPA utilizes transcriptome and proteomics to provide different protein maps, including tissue maps, cell maps, and pathology maps [14].

### Gene Expression Profiling Interactive Analysis (GEPIA)

GEPIA (http://gepia.cancer-pku.cn/) is a multidimensional cancer genome dataset that integrates massive data from the TCGA and GTEx. The website has various customized features, such as single gene analysis, tumor type analysis, and multigene analysis [15]. In our study, we performed differential expression analysis of TPM4 in gliomas as a validation set.

### Correlation between clinical features in glioma and TPM4 expression

We used the Xiantaoxueshu database (https://www.xiantao.love/writings) to evaluate the correlation between TPM4 expression and various clinical features. Patient age, WHO tumor grades, deletion of sequences at chromosomes 1p and 19q, mutations in the gene encoding isocitrate dehydrogenase (IDH), and responses to radiotherapy and chemotherapy were included among the clinical features to be evaluated.

### Survival analysis

Patients were divided into two groups according to the level of TPM4 expression. Based on the survminer package of R 3.6.3, we constructed a series of Kaplan–Meier (KM) survival curves to determine whether TPM4 expression levels affect clinical outcomes in patients with glioma.

### Univariate and multivariate regression analysis

We compared the OS and TPM4 expression levels between the two groups of glioma patients based on univariate and multivariate Cox regression analyses. We visualized the data using the ggplot2 package of R 3.6.3. TPM4 was statistically significant in Cox regression when *p* < 0.05.

### ROC analysis and construction of the nomogram

Time-dependent curve and nomogram model analyses of diagnoses were created using R packages, including rms packages, survival packages, timeROC packages, and ggplot2 packages. The clinical data we used for these analyses were acquired from the TCGA database.

### Functional enrichment analysis

We explored mRNA differential expression by using the DEseq2 package. False-positive results were corrected by the adjusted *P* value. We defined the screening thresholds for differentially expressed genes as follows: adjusted P < 0.05 and |log2 (fold change) | ≥ 2 (DEGs). Gene Ontology (GO) and Kyoto Encyclopedia of Genes and Genomes (KEGG) analyses were performed using clusterProfiler packages [16–18]. Visualization was performed using the ggplot2 package. An adjusted *P* < 0.05 was considered statistically significant in the enrichment results.

### Gene Set Enrichment Analysis (GSEA)

The mRNA expression of TPM4 was analyzed by R 3.6.3, followed by GSEA using the clusterprofiler and ggplot2 packages. P-adjusted < 0.05 and FDR (q value) < 0.25 were considered to be significantly enriched.

### Protein–Protein Interaction (PPI) comprehensive analysis

We used the STRING website (https://string-db.org) to investigate the protein–protein interaction (PPI) of TPM4-binding proteins. STRING is an online tool that constructs protein interactions [19, 20]. The main settings were as follows: active interaction sources (“text-mining and experiments”), the meaning of network edges (“evidence”), the minimum required interaction score [“Low confidence (0.400)”], and max number of interactors to show (“no more than 20 interactors”) operated. A Venn diagram was generated using the ggplot2 package to compare genes associated with TPM4 expression that interact with DEGs and TPM4. The expression of TPM4 was correlated with cross-analyzed genes using Spearman correlation analysis. *P* < 0.05 was considered statistically significant.

### TPM4 expression and immune infiltration

Single-sample gene set enrichment analysis (ssGSEA) was used to assess the large number of relevant immune cells infiltrating tumor tissue. We determined the level of immune cell infiltration in glioma by using the GSVA package of the immune dataset. The TISIDB (http://cis.hku.hk/TISIDB) is an integrated repository database that has a major function in discovering interactions between tumors and the immune system [21]. We further probed the immune relevance of TPM4 in glioma using this database. We used Spearman correlation analysis to determine the correlation between TPM4 expression and immune checkpoint gene levels.

### Statistical analysis

We performed all statistical analyses with R 3.6.3. The chi-square test was used to examine the relationship between TPM4 mRNA expression and clinical features. We analyzed the prognostic value of TPM4 mRNA expression by multivariate Cox analysis and Kaplan–Meier analysis. *P* < 0.05 represents statistical significance.

## Results

### TPM4 expression in different tumors and glioma patients

**Fig. 1:**
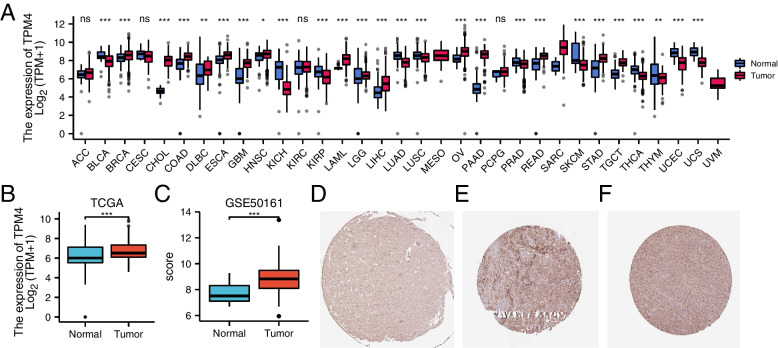
TPM4 expression status in tumors. TPM4 mRNA expression in different types of tumor tissues and normal tissues was based on the TCGA database and GTEx database (**A**). TPM4 mRNA expression in glioma tissues and normal tissues based on the TCGA database (**B**). TPM4 mRNA expression in glioma tissues and normal tissues based on the GEO database (**C**). Immunohistochemical staining of clinical normal cerebral cortex samples (**D**). Immunohistochemical staining of clinical LGG samples (**E**). Immunohistochemical staining of clinical GBM samples (**F**). *P < 0.05, **P < 0.01, and ***P < 0.001

**Fig. 2:**
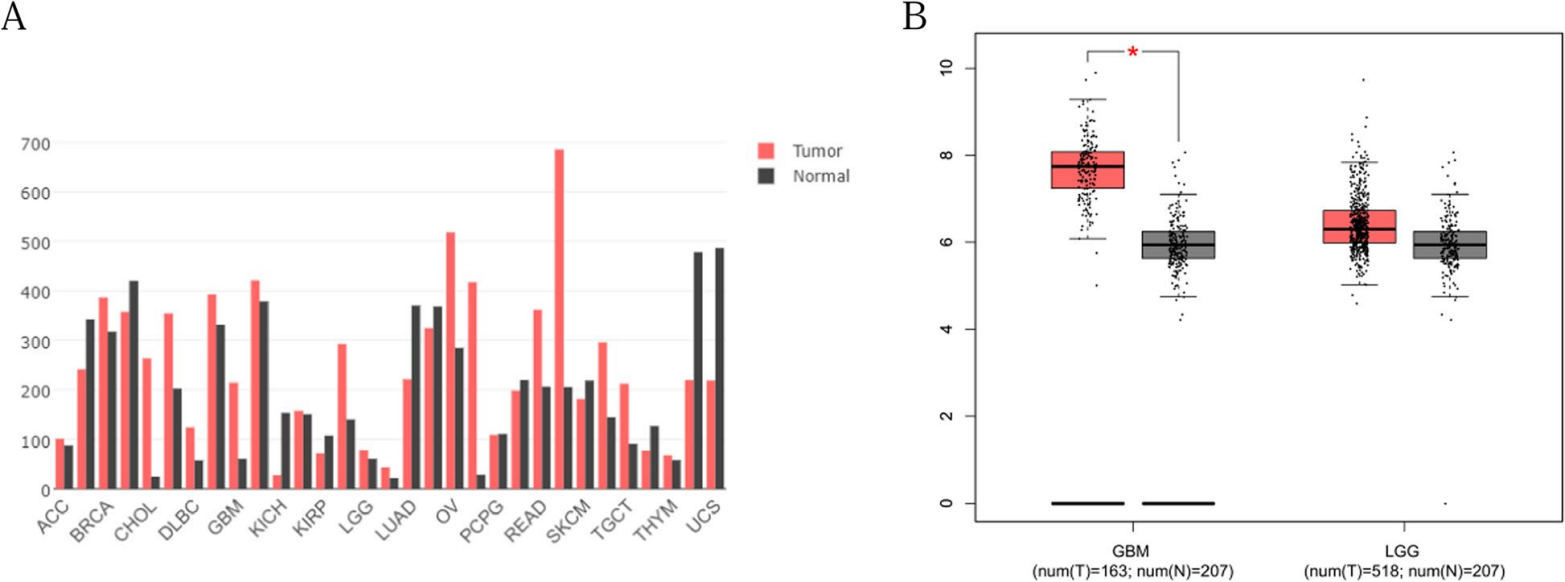
Expression of TPM4 in GEPIA. TPM4 expression across cancers (**A**). Differential expression of TPM4 between glioma and normal samples (**B**). *: *p*<0.05

First, we used the TCGA and GTEx databases to determine the expression of TPM4 in various tumor and normal tissue types. We found that the expression of TPM4 was significantly enhanced compared to normal tissues in breast invasive carcinoma (BRCA), lymphoid neoplasm diffuse large B-cell lymphoma (DLBC), esophageal carcinoma (ESCA), glioblastoma (GBM), lower-grade glioma (LGG), head and neck squamous cell carcinoma (HNSC), acute myeloid leukemia (LAML), liver hepatocellular carcinoma (LIHC), ovarian cancer (OV), pancreatic adenocarcinoma (PAAD), stomach adenocarcinoma (STAD) and testicular germ cell tumor (TGCT). In contrast, the expression of TPM4 was significantly lower than that in normal control tissues in bladder urothelial carcinoma (BLCA), kidney chromophobe (KICH), kidney renal papillary cell carcinoma (KIRP), lung adenocarcinoma (LUAD), skin cutaneous melanoma (SKCM), uterine corpus endometrial carcinoma (UCEC) and uterine carcinosarcoma (UCS) (Fig. 1A). Then, we analyzed TPM4 transcription levels in glioma by using TCGA and GEO data. We discovered that TPM4 mRNA was significantly upregulated in glioma tissues compared to healthy tissues (P < 0.001) (Fig. 1B-C). The protein levels of TPM4 in glioma were explored using the HPA (Fig. 1D-F). Immunohistochemical staining of glioma samples also confirmed that the level of TPM4 in tumor tissues was higher than that in adjacent normal tissues. In addition, in the GEPIA dataset, we compared the TPM4 mRNA expression level between glioma (including GBM and LGG) and normal samples. We found that there were higher gene expression levels of TPM4 in glioma than in healthy samples (Fig. 2A-B).

### Correlation between TPM4 expression and clinical characteristics

**Fig. 3:**
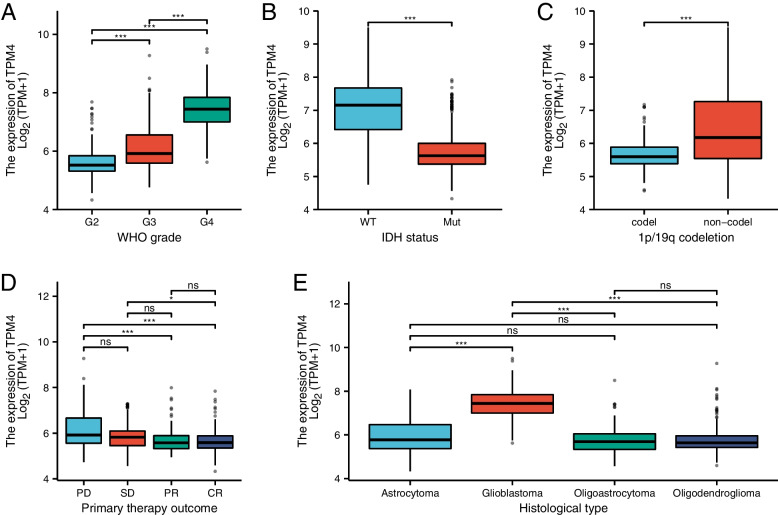
Box plot assessing TPM4 expression in patients with glioma according to different clinical characteristics. WHO grade (**A**), IDH status (**B**), 1p/19q codeletion (**C**), primary therapy outcome (**D**), and histological type (**E**)

We collected the characteristics of 696 patients with glioma from the TCGA database, including clinical and gene expression data. The patients were grouped into high and low TPM4 expression groups on the basis of the mean value of TPM4 expression (Table 1), following which putative correlations between TPM4 expression and clinical characteristics were evaluated using the rank test and logistic regression analysis. The results showed that high TPM4 expression was associated with a higher WHO classification (Fig. 3A). Furthermore, a comparison of TPM4 expression in gliomas with and without chromosome 1p and 19q codeletion and gliomas with wild-type and mutant IDH showed that TPM4 expression in gliomas without chromosome 1p/19q codeletion was higher and significantly higher in IDH-wild-type gliomas than in IDH-mutant gliomas (Fig. 3B-C). The expression level of TPM4 was significantly lower in patients with partial response (PR) and complete response (CR) to primary therapy than in patients with progressive disease (PD) (Fig. 3D). Moreover, we also observed that the level of TPM4 was higher in glioblastoma than in other histological types (Fig. 3E).

**Table 1:** The relationship between TPM4 mRNA expression and clinical characteristics in glioma

Characteristic	Low expression of TPM4	High expression of TPM4	p
n	348	348	
WHO grade, n (%)			< 0.001
G2	180 (28.3%)	44 (6.9%)	
G3	120 (18.9%)	123 (19.4%)	
G4	2 (0.3%)	166 (26.1%)	
IDH status, n (%)			< 0.001
WT	36 (5.2%)	210 (30.6%)	
Mut	311 (45.3%)	129 (18.8%)	
1p/19q codeletion, n (%)			< 0.001
codel	133 (19.3%)	38 (5.5%)	
noncodel	215 (31.2%)	303 (44%)	
Primary therapy outcome, n (%)			< 0.001
PD	53 (11.5%)	59 (12.8%)	
SD	90 (19.5%)	57 (12.3%)	
PR	49 (10.6%)	15 (3.2%)	
CR	106 (22.9%)	33 (7.1%)	
Age, n (%)			< 0.001
<=60	311 (44.7%)	242 (34.8%)	
>60	37 (5.3%)	106 (15.2%)	
Histological type, n (%)			< 0.001
Astrocytoma	112 (16.1%)	83 (11.9%)	
Glioblastoma	2 (0.3%)	166 (23.9%)	
Oligoastrocytoma	90 (12.9%)	44 (6.3%)	
Oligodendroglioma	144 (20.7%)	55 (7.9%)	
Age, median (IQR)	40 (32, 50.25)	53 (38, 63)	< 0.001

Univariate logistic regression analysis confirmed the association between high TPM4 expression and adverse clinicopathological features in glioma patients (Table 2). Univariate models showed that the expression of TPM4 was strongly correlated with WHO grade, primary therapy outcome, 1p/19q codeletion, IDH status, and histological type. Collectively, these results suggest that high TPM4 expression is significantly associated with glioma development.

**Table 2:** TPM4 expression associated with clinicopathologic characteristics (logistic regression)

Characteristics	Total(N)	Odds Ratio (OR)	*P* value
WHO grade (G3&G4 vs. G2)	635	9.691 (6.603-14.472)	<0.001
1p/19q codeletion (noncodel vs. codel)	689	4.933 (3.334-7.444)	<0.001
Primary therapy outcome (PR&CR vs. PD&SD)	462	0.382 (0.253-0.570)	<0.001
IDH status (Mut vs. WT)	686	0.071 (0.047-0.106)	<0.001
Histological type (Astrocytoma&Oligoastrocytoma&Oligodendroglioma vs. Glioblastoma)	696	0.006 (0.001-0.020)	<0.001

### TPM4 expression is independently associated with a poorer outcome in patients with glioma

**Fig. 4:**
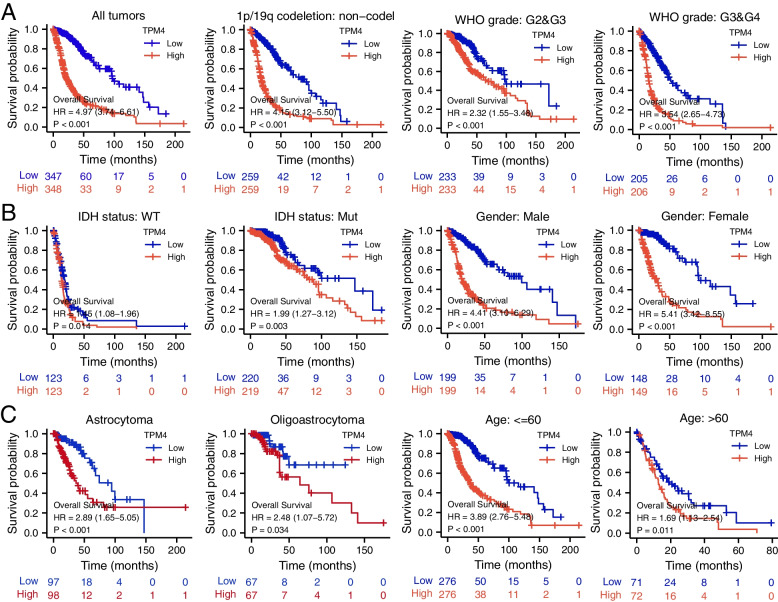
Analysis of the prognostic value of TPM4 in different glioma subgroups. Analysis of the prognostic value of TPM4 in different subgroups, including all tumors, 1p/19q status and WHO grade (**A**), IDH status and sex (**B**), histology type and age (**C**)

Kaplan–Meier analysis showed that high TPM4 mRNA expression was associated with poorer OS (overall survival) (*P* < 0.001). Assessment of the prognostic value of TPM4 expression in glioma patients subgrouped by histological type, sex, IDH mutation status, WHO grade, chromosome 1p/19q codeletion, and patient age showed that high expression of TPM4 was associated with poorer prognosis in all of these groups (Fig. 4A–C).

**Fig. 5:**
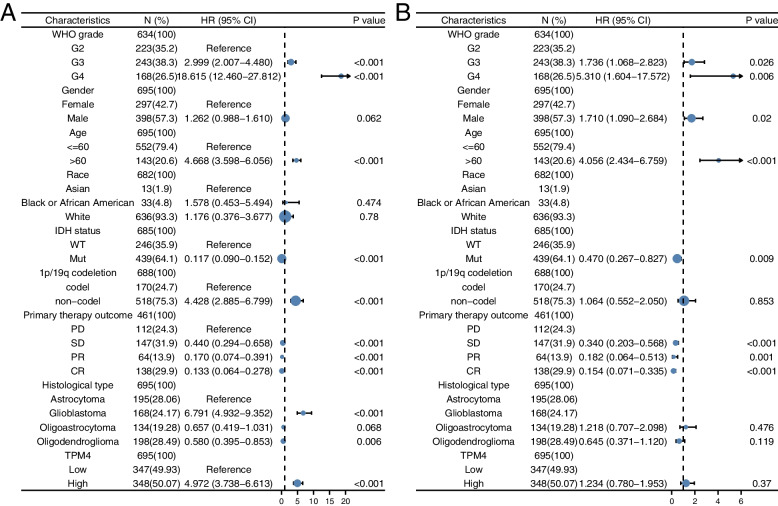
Univariate (**A**) and multivariate (**B**) regression analyses of TPM4 and other clinicopathologic parameters with OS in glioma patients

Univariate analysis indicated that TPM4 expression correlated with WHO grade, the age of patients, IDH status, chromosome 1p/19q codeletion, histological type, and primary therapy outcome (Table 3, Fig. 5). These results suggest that high TPM4 expression is associated with a poorer prognosis in gliomas.

**Table 3:** Univariate regression and multivariate survival model of prognostic covariates in patients with glioma

Characteristics	Total(N)	Univariate analysis		Multivariate analysis	
		Hazard ratio (95% CI)	*P* value	Hazard ratio (95% CI)	*P* value
WHO grade	634				
G2	223	Reference			
G3	243	2.999 (2.007-4.480)	**<0.001**	1.736 (1.068-2.823)	**0.026**
G4	168	18.615 (12.460-27.812)	**<0.001**	5.310 (1.604-17.572)	**0.006**
Gender	695				
Female	297	Reference			
Male	398	1.262 (0.988-1.610)	0.062	1.710 (1.090-2.684)	**0.020**
Age	695				
<=60	552	Reference			
>60	143	4.668 (3.598-6.056)	**<0.001**	4.056 (2.434-6.759)	**<0.001**
Race	682				
Asian	13	Reference			
Black or African American	33	1.578 (0.453-5.494)	0.474		
White	636	1.176 (0.376-3.677)	0.780		
IDH status	685				
WT	246	Reference			
Mut	439	0.117 (0.090-0.152)	**<0.001**	0.470 (0.267-0.827)	**0.009**
1p/19q codeletion	688				
codel	170	Reference			
noncodel	518	4.428 (2.885-6.799)	**<0.001**	1.064 (0.552-2.050)	0.853
Primary therapy outcome	461				
PD	112	Reference			
SD	147	0.440 (0.294-0.658)	**<0.001**	0.340 (0.203-0.568)	**<0.001**
PR	64	0.170 (0.074-0.391)	**<0.001**	0.182 (0.064-0.513)	**0.001**
CR	138	0.133 (0.064-0.278)	**<0.001**	0.154 (0.071-0.335)	**<0.001**
Histological type	695				
Astrocytoma	195	Reference			
Glioblastoma	168	6.791 (4.932-9.352)	**<0.001**		
Oligoastrocytoma	134	0.657 (0.419-1.031)	0.068	1.218 (0.707-2.098)	0.476
Oligodendroglioma	198	0.580 (0.395-0.853)	**0.006**	0.645 (0.371-1.120)	0.119
TPM4	695				
Low	347	Reference			
High	348	4.972 (3.738-6.613)	**<0.001**	1.234 (0.780-1.953)	0.370

**Fig. 6:**
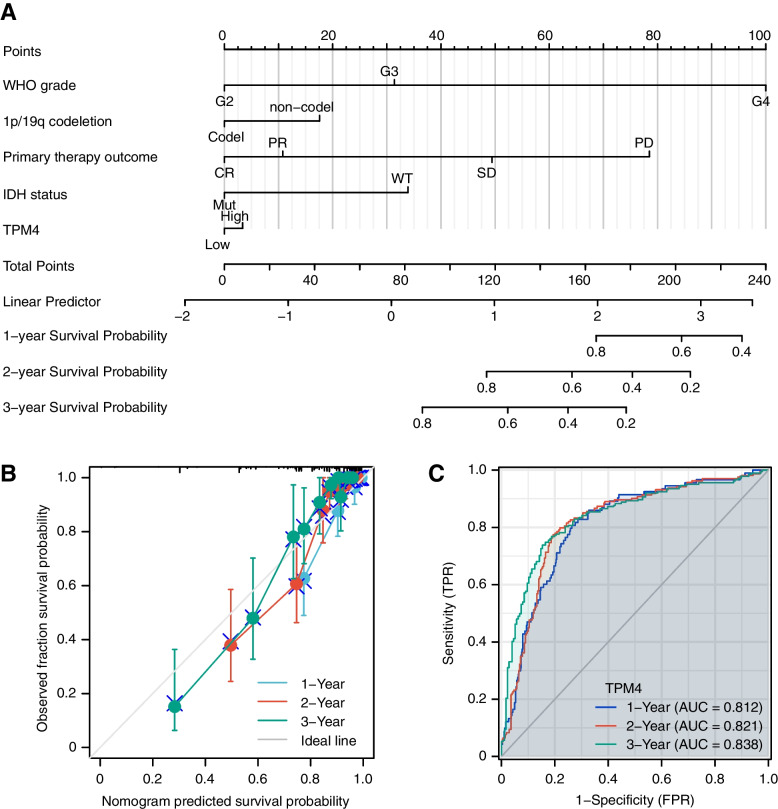
ROC analysis and construction and validation of the nomogram based on TPM4 expression. A nomogram for predicting the probability of 1-, 2- and 3-year OS in glioma patients (**A**). Calibration plots validate the efficiency of nomograms for OS (**B**). Time-dependent survival ROC curve analysis to predict 1-, 2- and 3-year survival rates (**C**)

Subsequently, we constructed a nomogram using WHO grade, chromosome 1p/19q codeletion, primary therapy outcome, IDH status, and TPM4 levels to predict 1-year OS, 2-year OS, and 3-year OS in patients with glioma (Fig. 6A). Then, calibration curves and time-dependent survival ROC curves ideally predicted nomograms of clinical outcomes at 1, 2, and 3 years (Fig. 6B-C). Taken together, TPM4 may be a useful biomarker for predicting OS in glioma patients.

### Functional inference of TPM4 in glioma

**Fig. 7:**
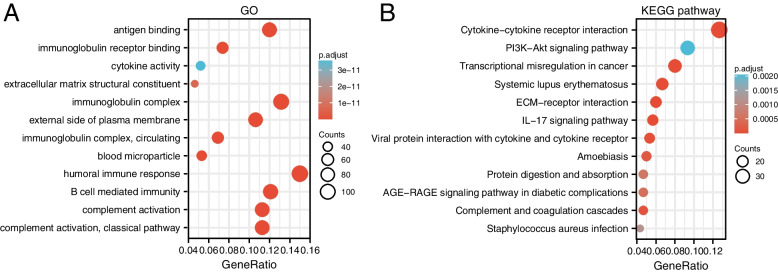
Enrichment analysis of TPM4 expression-correlated DEGs in glioma. GO enrichment (**A**) and KEGG enrichment analysis (**B**) of TPM4 expression-correlated DEGs

GO term annotation showed that coexpressed genes of TPM4 were mainly associated with the humoral immune response, immunoglobulin complex, antigen binding, external side of the plasma membrane, B-cell-mediated immunity, complement activation, immunoglobulin receptor binding, etc. (Fig. 7A). The KEGG pathway analysis indicated enrichment in cytokine–cytokine receptor interaction, PI3K-Akt signaling pathway, transcription misregulation in cancer, systemic lupus erythematosus, ECM-receptor interaction, and IL-17 signaling pathway, etc. (Fig. 7B).

### GSEA reveals TPM4-related signaling pathways

**Fig. 8:**
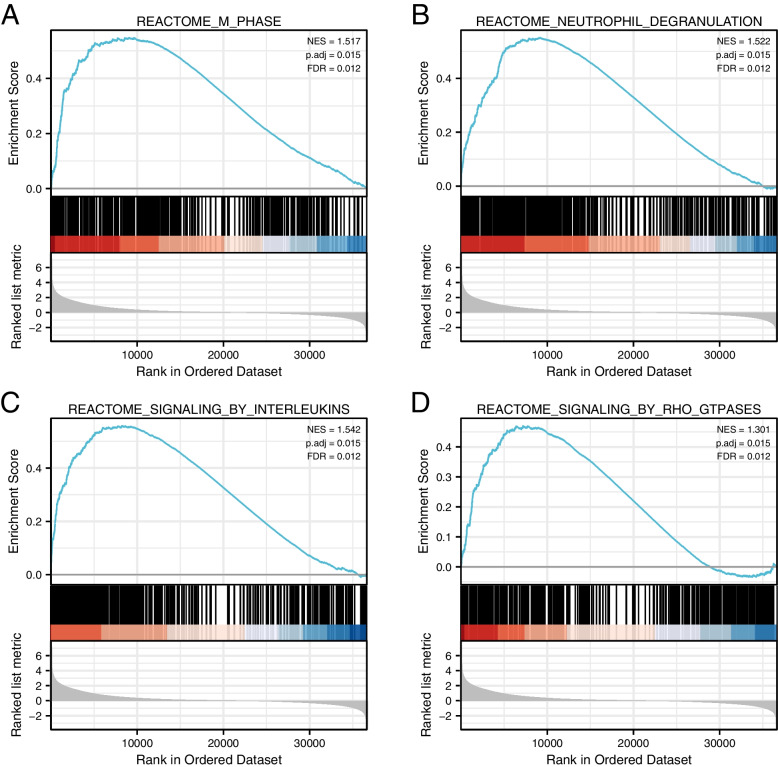
Enrichment plots by GSEA

We identified glioma-related signaling pathways using GSEA. The results indicated that the M phase of the cell cycle, neutrophil degranulation, signaling by interleukins, and signaling by rho GTPases were enriched and positively correlated with the TPM4 expression phenotype (Fig. 8A-D).

### Creating protein interaction networks

**Fig. 9:**
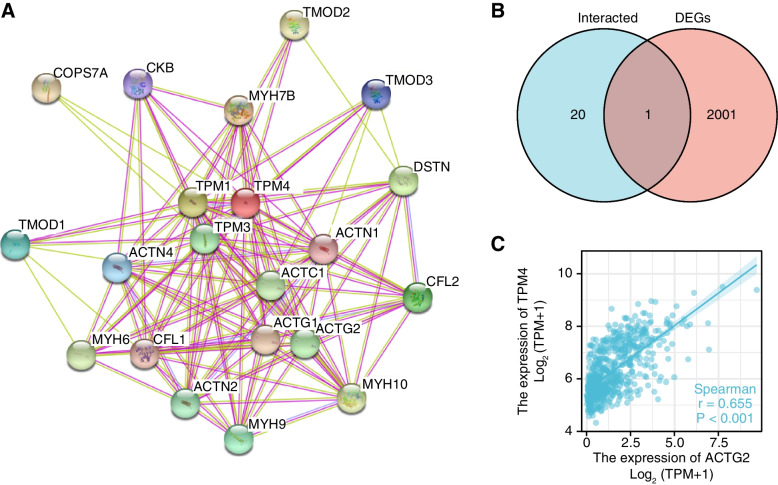
PPI network analysis of TPM4-related genes. The visualization of the interaction network of TPM4-binding proteins was obtained from the STRING database (**A**). An intersection analysis of TPM4 expression-correlated DEGs and TPM4-interacted genes was performed (**B**). Correlation analysis between TPM4 expression and screened common genes, including ACTG2 (**C**)

Interactions between proteins are crucial for the study of cancer metabolism and molecular mechanisms. Therefore, STRING was used to analyze the TPM4 protein PPI networks to determine their interactions in glioma progression. We visualized TPM4-binding protein interaction networks using text mining and experimental evidence identification (Fig. 9A). Moreover, by comparing the TPM4-interacting genes with the DEGs associated with TPM4 expression, their coexpressed gene ACTG2 was identified (Fig. 9B). Furthermore, there was a significant positive correlation between ACTG2 expression and TPM4 expression (*r* = 0.655, *p* < 0.001) (Fig. 9C).

### The expression of TPM4 in glioma patients correlates with the level of immune infiltration

**Fig. 10:**
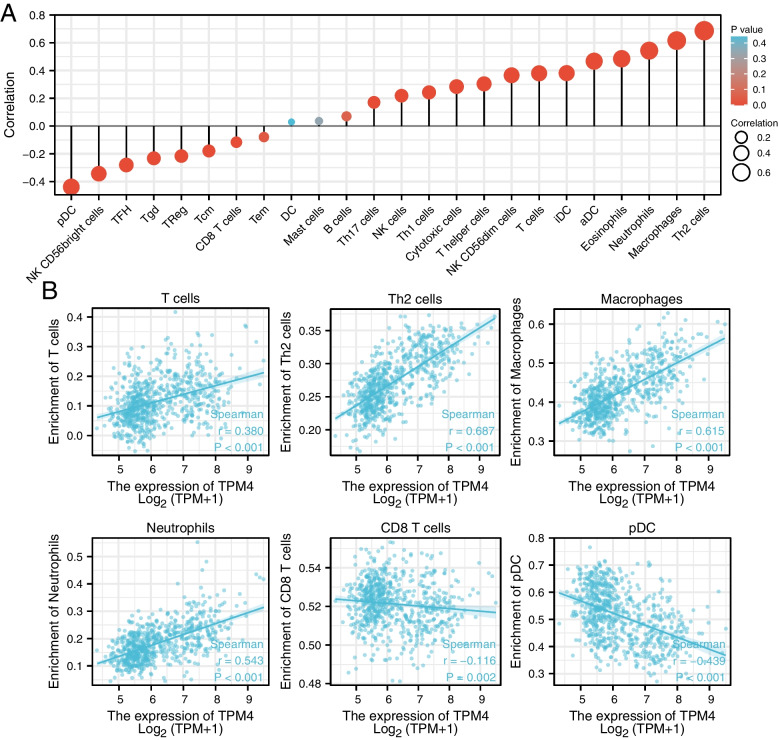
Correlation analysis of TPM4 expression with immune infiltration in glioma (**A**). The expression levels of TPM4 were positively correlated with the infiltration levels of T cells, Th2 cells, macrophages and neutrophils and negatively correlated with the infiltration levels of CD8 T cells and pDCs (**B**)

**Fig. 11:**
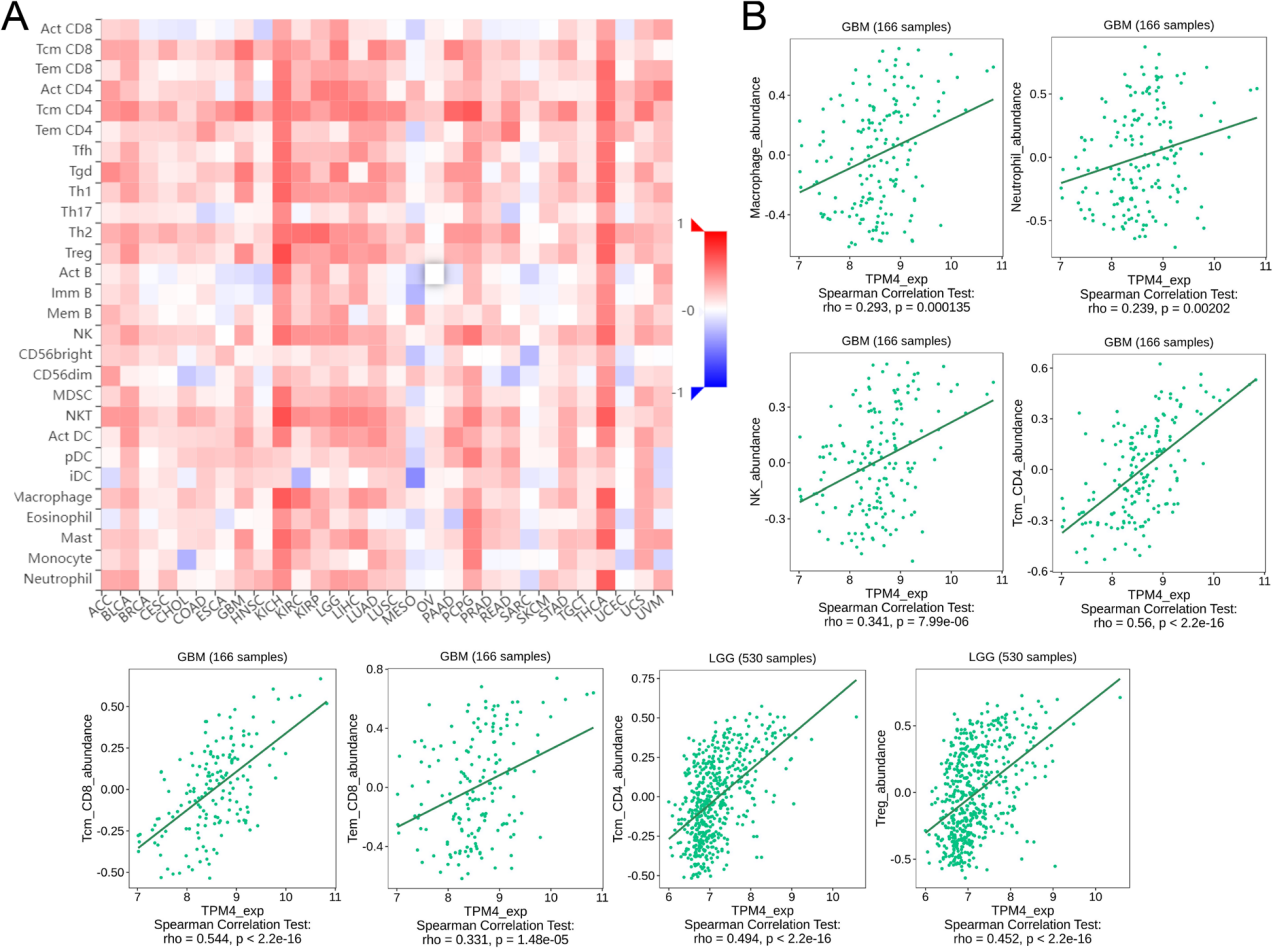
Correlation analysis of TPM4 expression with tumor-infiltrating lymphocytes (TILs) in cancer based on the TISIDB database. The landscape of the relationship between TPM4 expression and TILs in multiple types of cancers (red indicates a positive correlation, and blue indicates a negative correlation) (**A**). TPM4 expression was significantly positively associated with infiltrating levels of tcm_CD4, Treg, tcm_CD8, and tem_CD8 in glioma (**B**)

**Fig. 12:**
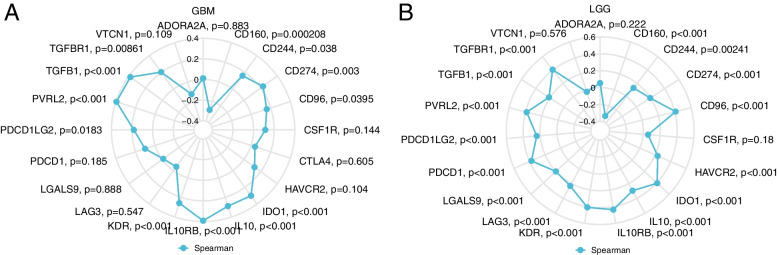
Correlation analysis of TPM4 expression with immune checkpoint genes. Correlation analysis of TPM4 expression levels with immune checkpoint gene levels in GBM (**A**). Correlation analysis of TPM4 expression levels with immune checkpoint gene levels in LGG (**B**)

We found that the expression of TPM4 in gliomas positively correlated with the infiltration of various immune cells, such as T cells, Th2 cells, macrophages, and neutrophils, while it negatively correlated with the infiltration of CD8 T cells and pDCs in glioma (Fig. 10). Subsequently, we explored the correlation between TPM4 expression and TILs using TISIDB. As shown in Fig. 11A, TPM4 expression correlated with TILs in different cancer types. Specifically, in glioma, many types of TILs were associated with TPM4 expression (Fig. 11B). A study showed that a new tumor immunotherapy strategy, immune checkpoint inhibitors (ICIs), could benefit patients with multiple cancers [22]. Next, we compared TPM4 expression with multiple immune control-related genes. We found that TPM4 is related to the molecular targets of glioma, such as CD160, IDO1, IL10, KDR, PVRL2, and TGFB1. This may be closely related to the mechanism by which high TPM4 expression leads to a worse prognosis in glioma (Fig. 12A-B).

## Discussion

Gliomas are the most common primary brain malignancies, and their highly malignant and aggressive growth can lead to severe clinical symptoms in patients, ultimately leading to death, reducing patients' quality of life, and increasing the burden on the health care system [1]. Current treatment strategies for glioma include traditional therapies, such as surgery and chemoradiotherapy, but the improvement in patient prognosis is limited. In the past decade, immunotherapy has emerged in the field of cancer treatment and has gradually become a promising treatment strategy [4, 23, 24]. However, not all patients respond well to this treatment. Therefore, we need to identify a new immune-related biomarker to aid in the treatment and prognosis of patients and investigate the underlying molecular mechanisms of glioma formation. Previous studies have shown that TPM4 is related to the development and progression of multiple cancers [8–12], but the study of TPM4 in glioma is not clear. To the best of our knowledge, our study is the first to use a public database to investigate the relationship between TPM4 as a clinical and immune biomarker of glioma.

In our study, we first determined the expression of TPM4 in whole WHO grade of glioma patients using the TCGA database. We discovered that TPM4 was consistently upregulated in whole-grade glioma samples compared with healthy normal samples. This result was further confirmed in the GSE50161 dataset and GEPIA database. Immunohistochemical results in the HPA database also demonstrated higher levels of TPM4 expression in gliomas compared to adjacent normal tissues at the protein level.

Next, we attempted to determine the expression level and prognostic value of the TPM4 gene in different clinical states of glioma. We discovered that high TPM4 expression was often related to worse clinicopathological features in gliomas. For example, we found that the higher the tumor grade of the glioma, the higher its TPM4 expression. Previous studies have shown that tumor cell heterogeneity increases with glioma tumor grade and negatively correlates with patient prognosis [25], which is consistent with our findings. In addition, we found that TPM4 expression was also related to IDH mutation status, chromosome 1p/19q codeletion, primary therapy outcome, and histological type in glioma patients. The Kaplan–Meier curve indicated that high TPM4 expression was closely associated with worse OS in glioma patients. We found that molecular features were closely related to glioma prognosis, in particular, chromosome 1p/19q codeletions and IDH mutation status had protective effects on patient prognosis. These two molecular features have been used as reference indicators for the degree of malignancy of gliomas in the 2016 WHO classification of gliomas, and their different statuses will lead to differences in patient prognosis [26, 27], which is consistent with our findings. This result shows that the chromosome 1p/19q codeletions and IDH mutation status are good indicators of glioma prognosis. These research findings strongly demonstrate that TPM4 may be a prognostic biomarker for glioma.

Next, we further verified that high TPM4 expression was an independent risk factor for glioma patients by Cox regression analysis. Moreover, the AUC values of the time-dependent survival ROC curve analysis were all higher than 0.8, all of which confirmed the clinical predictive value of TPM4. In addition, we used WHO grade, chromosome 1p/19q codeletion, primary therapy outcome, IDH status, and TPM4 levels as indicators to construct a prognostic nomogram, which can be employed by physicians to enhance the accuracy of the clinical identification of glioma patients.

The above findings confirm that TPM4 is a novel glioma oncogene and that its expression can significantly worsen the prognosis of glioma patients, but the molecular mechanism of its biological function needs to be further explored. GO/KEGG enrichment analysis proved that the relevant mechanism may involve the humoral immune response, immunoglobulin complex, antigen binding, B-cell-mediated immunity, complement activation immunoglobulin receptor binding, cytokine receptor interaction, external side of the plasma membrane, PI3K-Akt signaling pathway, transcription misregulation in cancer, systemic lupus erythematosus, ECM-receptor interaction, and IL-17 signaling pathway. We found that most of these mechanisms are immune-related. Furthermore, in our findings, a variety of signaling pathways, such as the PI3K-Akt signaling pathway, have been confirmed in previous studies [28–30]. We next performed GSEA and found that the potential mechanisms may involve the M phase of the cell cycle, neutrophil degranulation, signaling by interleukins, and signaling by rho GTPases, which may be potential mechanisms associated with poor prognosis in gliomas.

Considering our findings that multiple immune-related mechanisms are associated with TPM4 expression and glioma, we next explored the tumor microenvironment (TME). In addition to malignant cells, multiple components of the TME include fibroblasts, lymphocytes, tumor vasculature, dendritic cells, and cancer-associated fibroblasts [31, 32]. Previous studies have found that the development and infiltration of tumor cells depend on the complex tumor microenvironment, which plays an indispensable role in tumor growth and development [33, 34]. Immune cells, an important part of the TME, are critically involved in tumor survival and death. Our results demonstrate that TPM4 expression is related to multiple immune markers and levels of immune infiltration in glioma. Immune cell infiltration analysis revealed that high TPM4 expression in glioma was positively related to the levels of macrophages, neutrophils, and NK cells. These results were confirmed in the analysis of the TISIDB database. In addition, the radar map showed that TPM4 is closely related to various immune checkpoint genes, such as CD160, IDO1, IL10, KDR, PVRL2, and TGFB1. Immune checkpoint inhibitors (ICIs) have been shown to progressively improve the prognosis of patients with multiple cancers. ICIs are a new immunotherapy strategy that has transformed the treatment of a variety of cancers, including malignancies once thought to be incurable [35–38]. Many of the immune checkpoints identified in this study have been reported in previous studies, and many have been used in the clinical treatment of glioma [39–41]. Our results convincingly demonstrate that the TPM4 gene may play an important role in glioma immunity; therefore, TPM4-related research and novel targeted treatment may help improve the poor prognosis of glioma patients.

However, our study still has limitations. Since our data were all obtained from public databases, further experiments are required to validate our findings. In addition, due to data limitations, our study lacks the exploration of noninvasive tissues, which requires us to conduct further basic and clinical experiments in the future. It is worth mentioning that at the same time that our study is about to be completed, another study on members of TPM family in glioma has reached a similar conclusion to our study [42], and experimentally verified that TPM3, another member of TPM family, can be used as an independent prognostic factor for glioma.

## Conclusion

Overall, our study demonstrates that TPM4 can serve as a biomarker for glioma prognosis and diagnosis. We found that the expression of TPM4 was upregulated in gliomas, and high TPM4 expression was related to a worse prognosis in gliomas. We constructed a risk assessment model to help clinicians identify glioma patients. In addition, we found that TPM4 expression in glioma is closely associated with immunity, which provides a new direction and insight to promote the development of new immunotherapy strategies and treatment options for glioma patients.

## Data Availability

Publicly available datasets were analyzed in this study. Institutional review board approval was not required for our study because all databases are publicly available. These data can be found on the following website. TCGA: https://portal.gdc.cancer.gov/; GTEx: https://www.gtexportal.org/home/; GEO: https://www.ncbi.nlm.nih.gov/gds; HPA: https://www.proteinatlas.org/; GEPIA: http://gepia.cancer-pku.cn/; Xiantaoxueshu database: https://www.xiantao.love/writings; STRING: https://string-db.org; TISIDB: http://cis.hku.hk/TISIDB.

## Abbreviations

TPM4: Tropomyosin 4
WHO: World Health Organization
LGG: Low-grade glioma
GBM: Glioblastoma
TCGA: The Cancer Genome Atlas
GEO: Gene Expression Omnibus
GTEx: Genotype-Tissue Expression
HPA: Human Protein Atlas
GEPIA: Gene Expression Profiling Interactive Analysis
IDH: Isocitrate dehydrogenase
KM: Kaplan–Meier
GO: Gene Ontology
KEGG: Kyoto Encyclopedia of Genes and Genomes
PPI: Protein–protein interaction
ssGSEA: Single-sample gene set enrichment analysis
PR: Partial response
CR: Complete response
PD: Progressive disease
OS: Overall survival
ICIs: Immune checkpoint inhibitors
ROC: Receiver operating characteristic
TME: Tumor microenvironment
HR: Hazard ratio
